# Expression and characterization of the new antimicrobial peptide AP138L-arg26 anti *Staphylococcus aureus*

**DOI:** 10.1007/s00253-023-12947-w

**Published:** 2024-01-13

**Authors:** Kun Zhang, Na Yang, Da Teng, Ruoyu Mao, Ya Hao, Jianhua Wang

**Affiliations:** 1 Gene Engineering Laboratory, Feed Research Institute, Chinese Academy of Agricultural Sciences, No. 12 Zhongguancun Nandajie St., Haidian District, Beijing, 100081 People’s Republic of China; 2 Innovative Team of Antimicrobial Peptides and Alternatives to Antibiotics, Feed Research Institute, Chinese Academy of Agricultural Sciences, Beijing, 100081 People’s Republic of China; 3 Key Laboratory of Feed Biotechnology, Ministry of Agriculture and Rural Affairs, Beijing, 100081 People’s Republic of China

**Keywords:** AP138L-arg26, *Staphylococcus aureus*, Recombination expression, Safety, Bactericidal mechanism

## Abstract

**Abstract:**

The low activity and yield of antimicrobial peptides (AMPs) are pressing problems. The improvement of activity and yield through modification and heterologous expression, a potential way to solve the problem, is a research hot-pot. In this work, a new plectasin-derived variant L-type AP138 (AP138L-arg26) was constructed for the study of recombination expression and druggablity. As a result, the total protein concentration of AP138L-arg26 was 3.1 mg/mL in *Pichia pastoris* X-33 supernatant after 5 days of induction expression in a 5-L fermenter. The recombinant peptide AP138L-arg26 has potential antibacterial activity against selected standard and clinical Gram-positive bacteria (G^+^, minimum inhibitory concentration (MIC) 2–16 µg/mL) and high stability under different conditions (temperature, pH, ion concentration) and 2 × MIC of AP138L-arg26 could rapidly kill *Staphylococcus aureus* (*S. aureus*) (> 99.99%) within 1.5 h. It showed a high safety in vivo and in vivo and a long post-antibiotic effect (PAE, 1.91 h) compared with vancomycin (1.2 h). Furthermore, the bactericidal mechanism was revealed from two dimensions related to its disruption of the cell membrane resulting in intracellular potassium leakage (2.5-fold higher than control), and an increase in intracellular adenosine triphosphate (ATP), and reactive oxygen species (ROS), the decrease of lactate dehydrogenase (LDH) and further intervening metabolism in *S. aureus*. These results indicate that AP138L-arg26 as a new peptide candidate could be used for more in-depth development in the future.

**Key points:**

*• The AP138L-arg26 was expressed in the P. pastoris expression system with high yield*

*• The AP138 L-arg26 showed high stability and safety in vitro and in vivo*

*• The AP138L-arg26 killed S. aureus by affecting cell membranes and metabolism*

**Supplementary Information:**

The online version contains supplementary material available at 10.1007/s00253-023-12947-w.

## Introduction

As a major discovery in the twentieth century, antibiotics play an irreplaceable role in human health, animal epidemic prevention, and disease treatment (Coleman et al. 2015). However, due to the overuse of antibiotics, a variety of bacteria have developed drug resistance, including the intensively studied *Staphylococcus aureus* (*S. aureus*) through a variety of escape routes to avoid drug access, which can cause various animal diseases such as mastitis, endometritis, sepsis, and other systemic symptoms, seriously endangering the production of livestock and poultry and the development of animal husbandry (Coleman et al. 2015; Kim et al. 2019). Drug resistance was transmitted through the environment, humans, and livestock (Davies and Davies 2010), causing huge economic losses (El-Sayed Ahmed et al. 2020). Vancomycin is considered the last line of defense against infection by drug-resistant Gram-positive bacteria (G^+^), but resistance has quickly developed with the increase in drug use in clinical practice (Rao 1995). Therefore, there is an urgent need for an antibiotic substitution to address this issue.

Antimicrobial peptides (AMPs), as an important part of the natural immune barrier, exhibit antibacterial characteristics, immune regulation, and multiple other functions (Shinohara et al. 1995; Huttner and Bevins 1999; Peters et al. 2010; Wang et al. 2021a, b). The DBAASP database contains 20,523 antibacterial peptides and derivatives (Pirtskhalava et al. 2021),  being considered a rich resource for AMPs screening (Pinto et al. 2019). AMPs generally have the characteristics of cationic, hydrophobic, short sequence length (< 50aa), and unique three-dimensional (3D) structure including α-helix, β-sheet, and β-turn or coil; these play a vital role in the functional activity of AMPs for better understanding of the structure-activity relationship (Cao et al. 2015; Wang et al. 2018; Wang et al. 2021a, b; Wu et al. 2022). The AMPs, consisting of only amino acid in term of narrow definition, have multiple antibacterial mechanisms including destroying the cell membrane or wall or causing the contents to leak (Miao et al. 2016; Glukhov et al. 2008; Jhong et al. 2019; Lee et al. 2011; Ma et al. 2017); this is different from traditional heterocyclic peptide antibiotics, for which it is not easy to produce drug resistance. Therefore, they are becoming a research hotspot for the substitution of antibiotics (Gao et al. 2021; Pinto et al. 2019; Wu et al. 2021). Plectasin is a fungal defensin, isolated from *Pseudoplectania nigrella*, which can inhibit the synthesis of the bacterial cell wall by binding to the cell wall precursor Lipid II. It has a strong killing effect on G^+^ (Mygind et al. 2005; Schneider et al. 2010) and the ability to treat peritonitis and pneumonia caused by *Streptococcus pneumoniae* *and* *S.aureus* (Mygind et al. 2005). However, its high cost and low druggability limit its clinical applications. In order to solve the above problems, 12 kinds of plectasin-derived peptides were screened, analyzed, and verified in the DBAASP database (Zhang et al. 2014, 2021; Othman et al. 2018; Wang et al. 2021a, b; Huang et al. 2022), AP138 (DBAASPS_12115) created by Lociuro was predicted to be the best one among plectasin-derived peptides with high antimicrobial activity and a long postantibiotic effect (PAE) against G^+^ bacteria including *S. aureus* and methicillin-resistant *Staphylococcus aureus* (MRSA) (Lociuro et al. 2015; Groo et al. 2018). Compared with plectasin, there are three main differences in AP138: (i) Five amino acids, 9D, 13 M, 14Q, 17N, and 26 K, are mutated to 9S, 13L, 14R, 17R, and 26R, respectively; (ii) the positive charge increased from + 1 to + 4.5; (iii) the second structure α-helix (30.75 to 20.8%) and β-sheet (20.8 to 12.5%), β-turn (95.8 to 120.8%) changed obviously (Table 1 and S1). These studies and bioinformatics analysis all indicated that AP138 has research and development value in clinic therapeutics. However, AP138 is chemically synthesized due to D-type Arg at 26th site in sequence, so suffer from the heterologous expression difficulty and high cost (Koo and Seo 2019). Heterologous expressions may solve the problem of high cost, but the amino acid composition will be natural L-type amino acids (Lee et al. 2011; Lin et al. 2018). Therefore, the potential new different properties and mechanism of L-type AP138 (defined as AP138L-arg26 (DBAASPS_12115)) should be characterized as one of the major goals of this work (Groo et al. 2018; Pinto et al. 2019; Patel et al. 2009; Umerska et al. 2016; McEwen and Collignon 2018).

**Table 1:** Physicochemical properties and likelihood prediction of antimicrobial peptide

Peptide	Sequence	AMP probability	Hydrophobic ratio	Net charge +	GRAVY −	Hydrophobicity	Amphiphilicity index	Protein-binding potential	Molecular weight
Plectasin	GFGCNGPWDEDDMQCHNHCKSIKGYKGGYCAKGGFVCKCY	0.9495	32%	1	0.695	—	1.15	1.4	4407.99
AP138L-arg26	GFGCNGPWSEDDLRCHRHCKSIKGYRGGYCAKGGFVCKCY	0.992	33%	4.5	0.674	3.42	1.25	1.88	4460.125
NZ2114	GFGCNGPWNEDDLRCHNHCKSIKGYKGGYCAKGGFVCKCY	0.9895	33%	3.5	0.672	3.5	1.18	1.52	4417.049
NZX	GFGCNGPWSEDDIQCHNHCKSIKGYKGGYCARGGFVCKCY	0.9355	33%	2.5	0.5775	3.05	1.12	1.43	4389.981
MP1102	GFGCNGPWQEDDVKCHNHCKSIKGYKGGYCAKGGFVCKCY	0.88	33%	3.5	0.6475	4.47	1.24	1.28	4460.125
DLP4	ATCDLLSPFKVGHAACAAHCIARGKRGGWCDKRAVCNCRK	0.9455	50%	6.0	0.13	—	0.86	1.72	4275.26
ID13	ATCDLLSPFKVGHAACAAHCIARGKRGGWCDGRAVCNCRK	0.96	50%	5.5	0.0425	5.68	0.77	1.56	4203.96

The line means no results; all data in this table were predicted by online software (APD, DBAASP, and CAMP)

In this study, plectasin-derived peptide AP138L-arg26 was expressed in *Pichia pastoris* (*P. pastoris*) cells X-33 with a 5-L fermenter, and in vitro analysis of structure, antibacterial activity, stability, drug resistance, toxicity, and safety was performed. Finally, its bactericidal mechanism against *S. aureus* was revealed.

## Materials and methods

### Strains and cell lines

Gram-positive bacteria: *S. aureus* (ATCC 25923, 43300), *Staphylococcus epidermidis* (*S. epidermidis*) (ATCC 12228, 35984), and *Streptococcus agalactiae* (*S. agalactiae*) ATCC 13813 were purchased from American Type Culture Collection (ATCC). *S. aureus* CVCC 546 and *Streptococcus dysgalactiae* (*S. dysgalactiae*) CVCC 3938 were purchased from the China Veterinary Culture Collection Center (CVCC). *S. aureus* E48 was donated by Northwest Agriculture and Forestry University*. S. agalactiae* CAU-FRI-2022-01 and *S. agalactiae* CAU-FRI-2022-02 were donated by China Agricultural University. Gram-negative bacteria: *Escherichia coli* (*E. coli*) ATCC 25922 were purchased from ATCC. *Salmonella enteritidis* (*S. enteritidis*) CVCC 3377 and *Salmonella pullorum* (*S. pullorum*) CVCC1789 were purchased from CVCC. *Shigella flexneri* (*S. flexneri*) (CMCC 3926, 51571) were purchased from National Center for Medical Culture Collections (CMCC). *E. coli* O157 (CICC 21530), *Pseudomonas aeruginosa* (*P. aeruginosa*) (CICC 21625, CICC21630) were purchased from China Center of Industrial Culture Collection (CICC). *S. aureus* CAAS-FRI-2023-01 *and* CAAS-FRI-2023-02 were separated from Tianjin Aoxin Animal Husbandry Sheep Farm and Huanxian Sheep Farm, respectively. *E. coli* DH5α, *P. pastoris* X-33, and pPICZαA were purchased from Invitrogen (Beijing, China). RAW 264.7 mice macrophages were obtained from Peking Union Medical College. Bovine endometrial epithelial cell line BNCC35923 was purchased from by BeNa Culture Collection (Beijing, China).

### Reagents

The recombinant plasmid pPICZαA-AP138L-arg26 was synthesized by Sangon Biotech Co., Ltd. (Shanghai, China). Plasmid extraction kits, antibiotics (vancomycin and ceftiofur sodium), Dulbecco’s modified Eagle medium (DMEM), and fetal bovine serum (FBS) were purchased from Tiangen Co., Ltd, China Meilungel and Gibco (China), respectively. Other reagents were analytical grade.

### Model animal

The female ICR mice (SPF, 6–8 weeks, 20–25 g/mouse) were purchased from the Vital River Laboratories (VRL, Beijing, China). Mice acclimated to the environment for 1 week before the experiment. Animal experiments strictly complied with the requirements for animal handling and welfare of the Laboratory Animal Ethical Committee and its Inspection of the Feed Research Institute of Chinese Academy of Agricultural Sciences (CAAS) (AEC-CAAS-20090609).

## Biological information analysis of AP138L-arg26

AP138L-arg26 and plectasin derived AMP sequences were obtained from DBAASP database (https://www.dbaasp.org/home) (Pirtskhalava et al. 2021), their physical and chemical properties, 3D structure, and antimicrobial peptide possibility were predicted by APD database (https://aps.unmc.edu/AP/) (Wang et al. 2016), I-TASSER (https://zhanggroup.org/I-TASSER/) (Yang and Zhang 2015), and CAMP database (http://www.camp.bicnirrh.res.in/), respectively (Waghu et al. 2014).

## Expression, purification, and identification of AP138L-arg26

The nucleic acid sequence of AP138L-arg26 (GenBank ID: 2736833) was subjected to codon preference using the Reverse Translate Tool (http://www.bioinformati cs.org/sms2/rev_trans.html), then a Kex2 signaling peptide cleavage site was added at its N-terminus (Figure S1) and inserted into the eukaryotic expression vector pPICZαA between the double enzyme (*Xho*I and *Xba*I) cleavage site, to construct the plasmid pPICZαA-AP138L-arg26*.* Additionally, the pPICZαA-AP138L-arg26 was digested using *Pme*I and transformed into competent *P. pastoris* X-33 cells via electroporation. Peptide purification was carried out with the AKTAxpress system. Expression of AMP AP138L-arg26 was identified using Tricine-SDS-PAGE firstly, and the purified AP138L-arg26 was identified using MALDI-TOF/TOF MS (Ultraflextreme, Bruker, Germany), finally, the concentration was detected using Bradford assay kits.

## Antimicrobial activity and pharmacodynamic analysis in vitro

### Minimum inhibitory concentration (MIC) and minimum bactericidal concentration (MBC)

To determine the MIC values of AP138L-arg26, we used the broth microdilution technique as previously described (Andrews. 2001; Wiegand et al. 2008). In brief, AP138L-arg26 peptide were diluted from 1280 to 2.5 µg/mL and added to a 96-cells plate with 10 µL per well. The bacteria in the logarithmic stage were diluted to 10^5^ CFU/mL with 90 µL per well. All plates were cultured in a 37 °C constant-temperature incubator; no visible growth of bacteria was the MIC after 18 h incubation. The MBC method was also based on the CLSI 2021 guidelines. In brief, after the bacteria were incubated at 1 × , 2 × , 4 × , and 8 × MIC of AP138L-arg26, the cultures were coated with Mueller-Hinton Agar (MHA) plates, the concentrations of AP138L-arg26 with 99.99% the bacteria killed was defined as MBC.

### Time-killing curve assay

The time-killing curve of AP138L-arg26 against MRSA ATCC 43300 was used to analyze the pharmacodynamics. The method was based on previous laboratory experiments (Flamm et al. 2019; Yang et al. 2019). Briefly, exponential-phase bacteria were diluted to 1 × 10^5^ CFU/mL, and peptides underwent a twofold gradient dilution setting of 4 × , 2 × , and 1 × MIC. There was incubation in a 37 °C thermostatic shaker at 200 rpm for 0–24 h to collect samples, which were plated on MHA plates. Colony numbers were recorded at all collected time points.

### PAE assay

The method of PAE of AP138L-arg26 against MRSA ATCC 43300 or *S. aureus* CVCC 546 was described previously (Wang et al. 2018). The formula is PAE = *T* − *C* (*T* is the time required for the number of colonies in the sample treatment group to increase by tenfold, and *C* is the time required for the untreated group).

### Intracellular antibacterial activity

The method of intracellular bactericidal effect was described previously (Wang et al. 2018). Only the number of cells and bacteria changed from 2.5 × 10^5^ to 2 × 10^5^ cells/mL.

## Synergism with antibiotics

### Fractional inhibitory concentration index (FICI)

The synergistic effects of AP138L-arg26 with different antibiotics were evaluated using a checkerboard assay. The synergistic effect was calculated using the FICI as follows: FICI = FIC of AP138L-arg26 + FIC of antibiotic; FIC = MIC_c_/MIC_a_, where MIC_c_ is the MIC of the peptide and antibiotic in combination, and MIC_a_ is the MIC of the peptide/antibiotic alone (Blier et al. 2010). Three parallel experiments were performed for each group. The efficacy of combination therapy was defined as FICI ≤ 0.5, 0.5 < FICI ≤ 1, 1 < FICI ≤ 4, and FICI > 4 indicating synergy, addition, no difference, and antagonism, respectively.

### Growth curve of *S. aureus*

Samples were taken during the logarithmic growth of *S. aureus* and diluted to 1 × 10^5^ CFU/mL (Delpech et al. 2019). Different concentrations of AP138L-arg26, ceftiofur sodium, or a combination of them with an equal volume of bacteria were added to a 96-well microplate. The growth curves were recorded using a fully automatic growth curve recording instrument.

## Stability analysis

### Artificial gastric and intestinal juice stability

AP138L-arg26 was incubated in artificial gastric and intestinal juice (Beijing Coolaber Technology Co., Ltd., Beijing, China) for 0.083, 0.167, 0.25, 0.75, and 1 h. The antimicrobial activity of AP138L-arg26 against MRSA ATCC 43300 was tested through the MIC assay.

### Thermal, pH, and salt stability

The thermal, pH, and salt stability of AMPs were studied previously (Zhang et al. 2011). Briefly, the AP138L-arg26 was incubated at different temperatures (4/20/40/60/80/100 °C), pH values (2/4/6/8/10), and kinds of salt ions (50/100/150/200/300 mM NaCl, 1.25/2.5/5 mM KCl, MgCl_2_, CaCl_2_) for 1 h at 37 °C, respectively. The antimicrobial activity of AP138L-arg26 against MRSA ATCC 43300 was tested using MIC assay.

## Circular dichroism (CD) spectrum assay

The secondary structures of AP138L-arg26 were detected using CD (Bio-Logic MOS450 spectropolarimeter, France) in a simulated eukaryotic cell environment (50% trifluoroethanol, TFE) and bacterial membrane environment (20 mM sodium dodecyl sulfate, SDS) (Yao et al. 2018).

## Safety evaluation in vitro and in vivo

### Hemolysis activity

The hemolysis of AP138L-arg26 to fresh ICR mouse erythrocytes was described previously (Zheng et al. 2021). Briefly, 8% of mouse erythrocytes were incubated with different concentrations of antimicrobial peptide AP138L-arg26 (256, 128, 64, 32, 16, 8, 4, 2, 1, 0.5 µg/mL) in equal volumes. The blank and positive control was phosphate buffered saline (PBS) and 0.1% Triton X-100, respectively.

### Cytotoxicity

The cytotoxicity of AP138L-arg26 to mouse macrophages RAW264.7 was detected by the thiazolyl blue tetrazolium bromide (MTT) method as described previously (Shen et al. 2021).

### Acute toxicity in mice

AP138L-arg26 was administrated by intraperitoneal injection (*n* = 4 per group, 10 mg/kg, body weight 25 g) every day for 1 week (Shi et al. 2022). After 7 days, the mice were euthanized to collect anticoagulant whole-blood and tissues (liver, spleen, kidney, and lung), which were used for whole blood and biochemical detection, or tissue sections (hematoxylin-eosin (HE) staining), respectively.

## Antimicrobial mechanism of peptides

### Scanning electron microscopy (SEM) assay

The effect of peptides on changes in bacterial morphology was observed by scanning electron microscopy. Briefly the exponential phase *S. aureus* CVCC 546 cells (1 × 10^9^ CFU/mL) were incubated with 2 × MIC peptides at 37 °C for 0.5 h, 1 h, and 2 h. The untreated bacteria were the negative control. The processing methods and steps of the samples were described in detail in our previous study (Li et al. 2020). Briefly, there were two important steps: (1) Sample preparation: incubation, cleaning, fixation, drying, gold spraying. (2) Observation using microscope. Only one concentration was used to deal with bacteria at different times (0.5, 1, and 2 h).

### SYTO9/propidium iodide (PI) assay

To explore the disruption of bacterial cell membranes by the AP138L-arg26, a SYTO9/PI kit was used to detect the integrity of cell membranes (L7007 LIVE/DEAD^R^ BacLight™ Bacerial Viability Kits). The unique feature of this kit is that SYTO9 alone can pass through integral cell membranes, while PI can only penetrate damaged cell membranes. Briefly, the *S. aureus* CVCC 546 cells (1 × 10^9^ CFU/mL) were incubated with AP138L-arg26 (2 × MIC, 37 °C for 1 h), and a volume of 3 µL SYTO9/PI (1.5 µL: 1.5 µL) was added to 1 mL of bacterial suspension. Finally, the results were observed using fluorescence microscopy.

### Membrane fluidity assay

The final concentration of 10 µM Laurdan was used to detect the effect of AP138L-arg26 on bacterial cell membrane fluidity (Shi et al. 2022). Briefly, (1) co-incubation of bacteria with Laurdan; (2) the stained bacteria are co-incubated with the peptide; (3) spectrophotometer (Tecan, Männedorf, Switzerland) detection. The calculation formula: generalized polarization (GP) = (I435 − I490)/(I435 + I490).

### Membrane depolarization assay

To further explore the effect of AP138L-arg26 on the bacterial membrane, the membrane probe DiSC_3_(5) was selected to detect the change in membrane potential (Wang et al. 2020). Briefly, *S. aureus* was washed and resuspended in PBS to 1 × 10^8^ CFU/mL and incubated with 0.5 mM membrane dyes DiSC_3_(5) at 37 °C for 1 h in the dark. Then, 90 µL of stained bacterial suspension and 10 µL of AP138 L-arg26 (1 × , 2 × , 4 × MIC) were mixed and added to black and clean 96-cell plates and the fluorescence intensity was determined using a spectrophotometer (excitation wavelength 622/emission wavelength 670 nm, Infinite M200).

### Potassium ion (K^+^) leakage

The integrity effect of antimicrobial peptide AP138L-arg26 on bacterial cell membranes was further verified, and the K^+^ leakage assay was previously described (Li et al. 2020). Briefly, the main procedures were as follows: Firstly, prepare the *S. aureus* CVCC 546 bacteria suspension at a concentration of 1 × 10^8^ CFU/mL. Secondly, incubate the bacterial suspension with 2 × MIC AP138L-arg26 at different times (15/30/60/90/120 min) at 37 °C, with untreated cells and nisin used as negative and positive controls. Finally, the supernatants were detected using inductively coupled plasma mass spectrometry (ICP-MS) (SantaClara, CA, USA).

### Intracellular adenosine triphosphate (ATP) determination

As ATP is the most direct source of energy for living organisms, the effect of antimicrobial peptides on ATP was explored with an ATP assay kit (Beyotime, Shanghai, China). Briefly, exponential-stage *S. aureus* CVCC 546 (1 × 10^8^ CFU/mL) were incubated with different concentrations of AP138L-arg26 (1 × , 2 × , 4 × MIC) for 1 h, and the pellets were collected, lysed, and centrifuged to harvest the intracellular supernatant. The luminescence was detected using an Infinite M200 Microplate reader (Tecan, Luminescence signals).

### Lactic dehydrogenase (LDH) activity

As disruption of the cell membrane structure leads to the release of LDH from the cytoplasm into the culture medium, the detection of LDH in bacteria can further reveal the antibacterial mechanism (Shi et al. 2022). Briefly, *S. aureus* cells were co-incubated with AP138L-arg26 for 6 h. Later, the pellets were collected and sonicated (3 s/10 s, 30 times). The intracellular LDH activity was detected using an Infinite M200 Microplate reader (Tecan, Luminescence signals). The calculated results of LDH% = treated cells/the control.

### Reactive oxygen species (ROS) measurements

The probe 2′,7′-Dichlorodihydrofluorescein diacetate (DCFH-DA) was used to measure the level of ROS in bacteria after incubated with AP138L-arg26 (Wang et al. 2021a, b; Shi et al. 2022). Briefly, exponential stage bacteria were incubated with DCFH-DA (10 µM, 37 °C, 0.5 h), and the stained bacteria were treated with AP138L-arg26 (1 × , 2 × , 4 × MIC) for 1 h. The ROS were detected at excitation wavelength (488 nm) and emission wavelength (525 nm) using a microplate reader (Tecan, Männedorf, Switzerland).

## Statistical analysis

The software GraphPad Prism (version 8, USA) was used to analyze all data, and ANOVA was the method to determine the statistical significance. The results are presented as means ± standard deviation (SD). A *P* value of < 0.05 was considered statistically significant.

## Results

### Analysis of AP138 and designation of AP138L-arg26

**Fig. 1:**
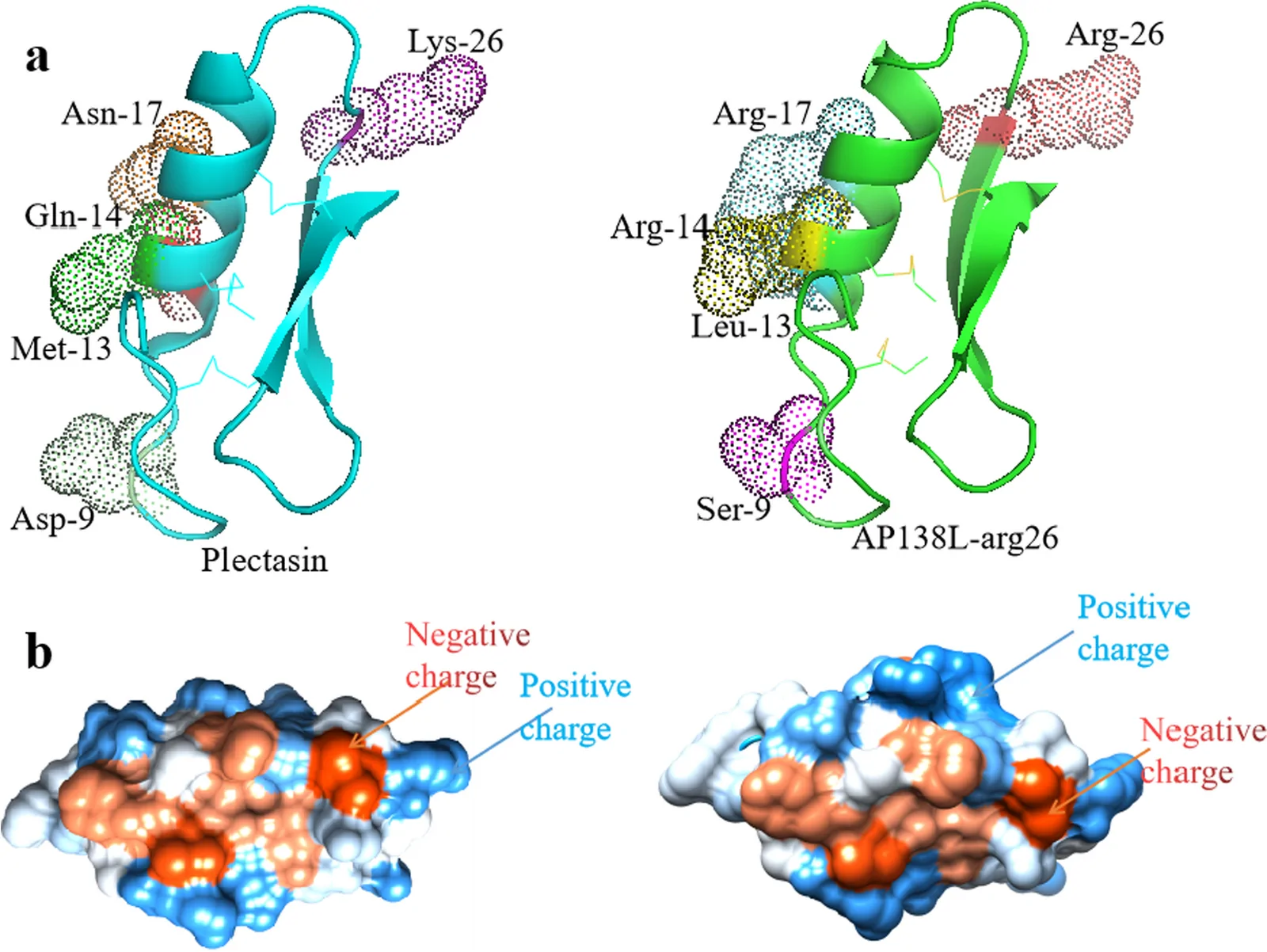
The 3D structure analysis of plectasin (left) and AP138L-arg26 (right). **a** Molecular modeling of plectasin and AP138L-arg26. **b** Electrostatic surface of plectasin (left) and AP138L-arg26 (right). Blue and red represent positive and negative charge, respectively

Plectasin was searched for as a keyword in the DBAASP database, and 12 sequences were retrieved; these derived peptides, AP138, NZ2114, NZX, MP1102, and MP1106, and other reported peptides DLP4, ID3, and P2 were also collected. Their physicochemical properties are shown in Table 1. AP138L-arg26 (GFGCNGPWSEDDLRCHRHCKSIKGYR_L26_G GYCAKGGFVCKCY) was predicted to have the highest AMP possibility (0.992) and charge (+ 4.5) (Table 1) and so was considered the plectasin-derived peptide. Through secondary and 3D structure analysis, we found that AP138L-arg26 conformation changed greatly with reduced α-helix/β-sheet and increased β-turn compared with the parent peptide plectasin (Table 1). Meanwhile, the electron cloud of amino acids at the 9th, 13th, 14th, 17th, and 26th positions changed significantly with the mutant amino acids; especially at the 13th, 14th, and 17th positions, more than 2 electron clouds changed at α-Helix, which may affect the function of peptides (Table 1 and Fig. 1).

### Design, expression, and purification of AP138L-arg26

**Fig. 2:**
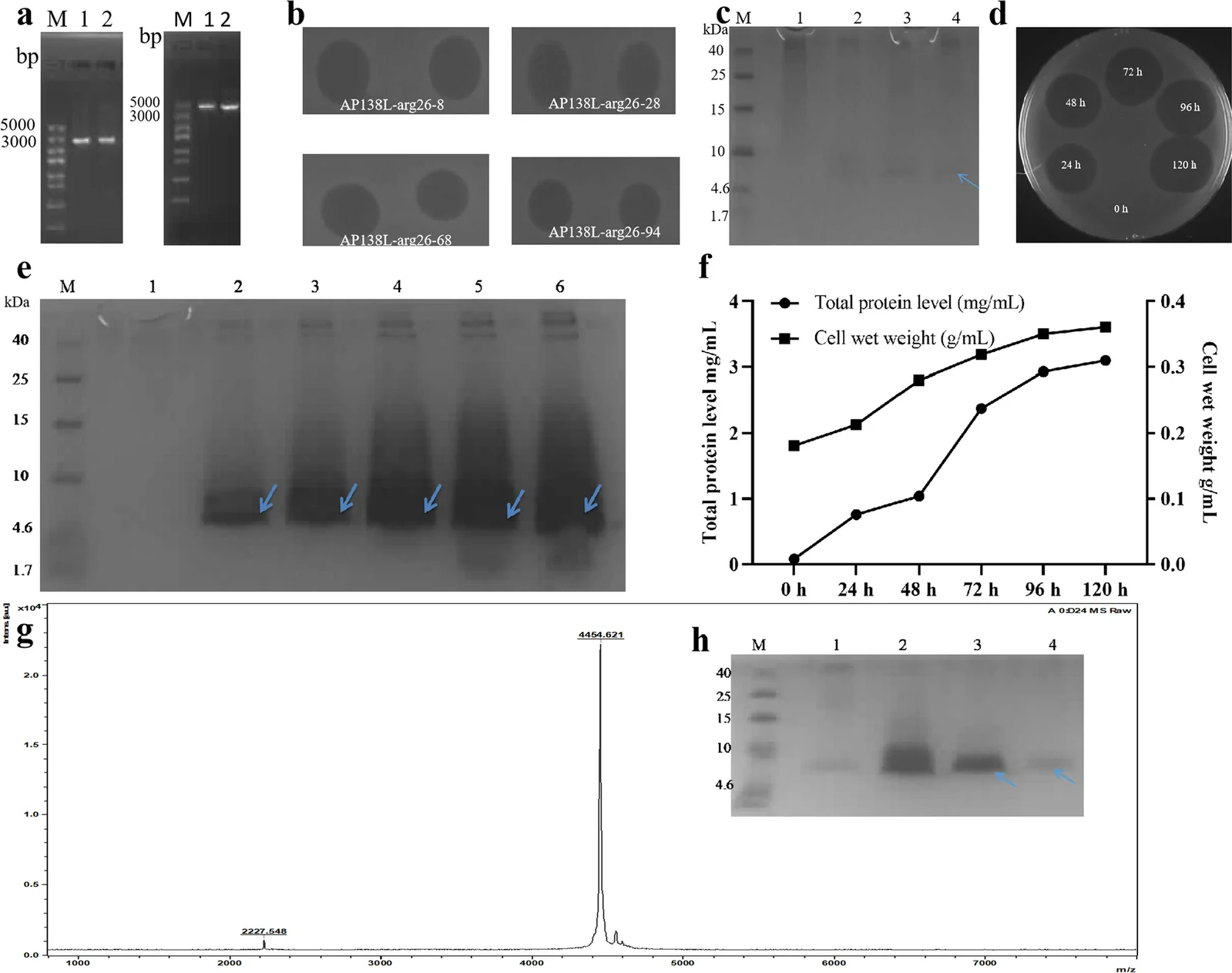
The construction of the pPICZαA-AP138L-arg26 plasmid and the expression of AP138L-arg26 in *P. pastoris* X-33 at the shaking flask and fermenter level. **a** Recombinant plasmids pPICZαA-AP138L-arg26 and linearized gel electrophoresis, lanes 1–2 were pPICZαA-AP138L-arg26 (left) and linearized plasmid. **b** Screening better AP138L-arg26 positive transformants AP138L-arg26-8, AP138L-arg26-28, AP138L-arg26-68, and AP138L-arg26-94 by inhibition zone against *S. aureus* ATCC 43300. **c** Tricine-SDS-PAGE analysis of AP138L-arg26 expression in shaking flask level, lanes 1–4 were fermentation supernatants of induction at 0 h, 24 h, 48 h, and 96 h, respectively. **d** Antimicrobial activity against *S. aureus* ATCC 43300 of AP138L-arg26 in fermenter level was analyzed by inhibition zone assay at 0 h, 24 h, 48 h, 96 h, and 120 h, respectively. **e** Tricine-SDS-PAGE analysis of AP138L-arg26 expression in fermenter level, lanes 1–6 were fermentation supernatants of induction at 0 h, 24 h, 48 h, 96 h, and 120 h, respectively. **f** Time curves of the total protein levels and cell wet weights at 0 h, 24 h, 48 h, 96 h, and 120 h, respectively. **g** Tricine-SDS-PAGE analysis of AP138L-arg26 purification. **h** MALDI-TOF MS analysis of the purified AP138L-arg26

The recombinant plasmid pPICZαA-AP138L-arg26 was linearized and transferred into *P. pastoris* X-33 competent cells. The recombinant plasmid sequence length was 3000–5000 bp (Fig. 2a). The positive transformants were screened using the inhibition zone test, and transformants AP138L-arg26-8, AP138L-arg26-28, AP138L-arg26-68, and AP138L-arg26-94 had a good antibacterial effect with a large and clear inhibition zone (Fig. 2b). Tricine-SDS-PAGE analysis of AP138L-arg26 expression in shaking flask level showed that the protein band was around 4.6 kDa, which was consistent with the predicted value of 4.46 kDa (Fig. 2c). The positive transformants AP138L-arg26 with the highest effect (AP138L-arg26-8) was chosen and expressed with a 5-L fermenter, which displayed high production compared with the shaking flask (Fig. 2d, e). The total protein of fermentation supernatant was 3.1 mg/mL and the biomass was 0.36 g/mL after induction in a 5-L fermenter at 120 h (Fig. 2f). The peptide was purified using the cation-exchange column (AKTAxpress system), and only a target peptide was detected at around 4.6 kDa through Tricine-SDS-PAGE band and a single peak was detected in matrix-assisted laser desorption/ionization time-of-flight mass spectrometry (MALDI-TOF/TOF MS) of 4464.21 Da (Fig. 2g, h).

### MICs and MBCs

The results of MICs and MBCs showed that AP138L-arg26 had potent antimicrobial activity against G^+^ bacteria such as *S. aureus* (2–16 µg/mL), *Streptococcus* (4 µg/mL), and *S. epidermidis* (4 or 8 µg/mL) with MIC values of 4–16 µg/mL, and the MBCs were around fourfold higher than MICs (Table 2). Antimicrobial activity was slightly lower than AP138 (Groo et al 2018) and vancomycin, but AP138L-arg26 retained good activity. These results show that AP138L-arg26 has potential for drug development against G^+^.

**Table 2:** The MIC and MBC of AP138L-arg26 and vancomycin

Strain	MIC (µg/mL)		MBC (µg/mL)	
	AP138L-arg26	Van	AP138L-arg26	Van
*Staphylococcus aureus* ATCC 25923	16	1	32	2
*Staphylococcus aureus* ATCC 43300	8	1	8	1
*Staphylococcus aureus* E48	2	1	8	8
*Staphylococcus aureus* CVCC 546	4	1	32	1
*Staphylococcus aureus* CAAS-FRI-2023-01	4	1	16	2
*Staphylococcus aureus* CAAS-FRI-2023-02	8	2	32	8
*Staphylococcus epidermidis* ATCC 12228	4	2	16	4
*Staphylococcus epidermidis* ATCC 35984	8	2	16	4
*Streptococcus dysgalactiae* CVCC 3938	4	2	32	2
*Streptococcus agalactiae* ATCC 13813	4	2	8	4
*Streptococcus agalactiae* CAU-FRI-2022-01	4	2	32	8
*Streptococcus agalactiae* CAU-FRI-2022-02	4	2	32	4

### Time killing curves and PAE

**Fig. 3:**
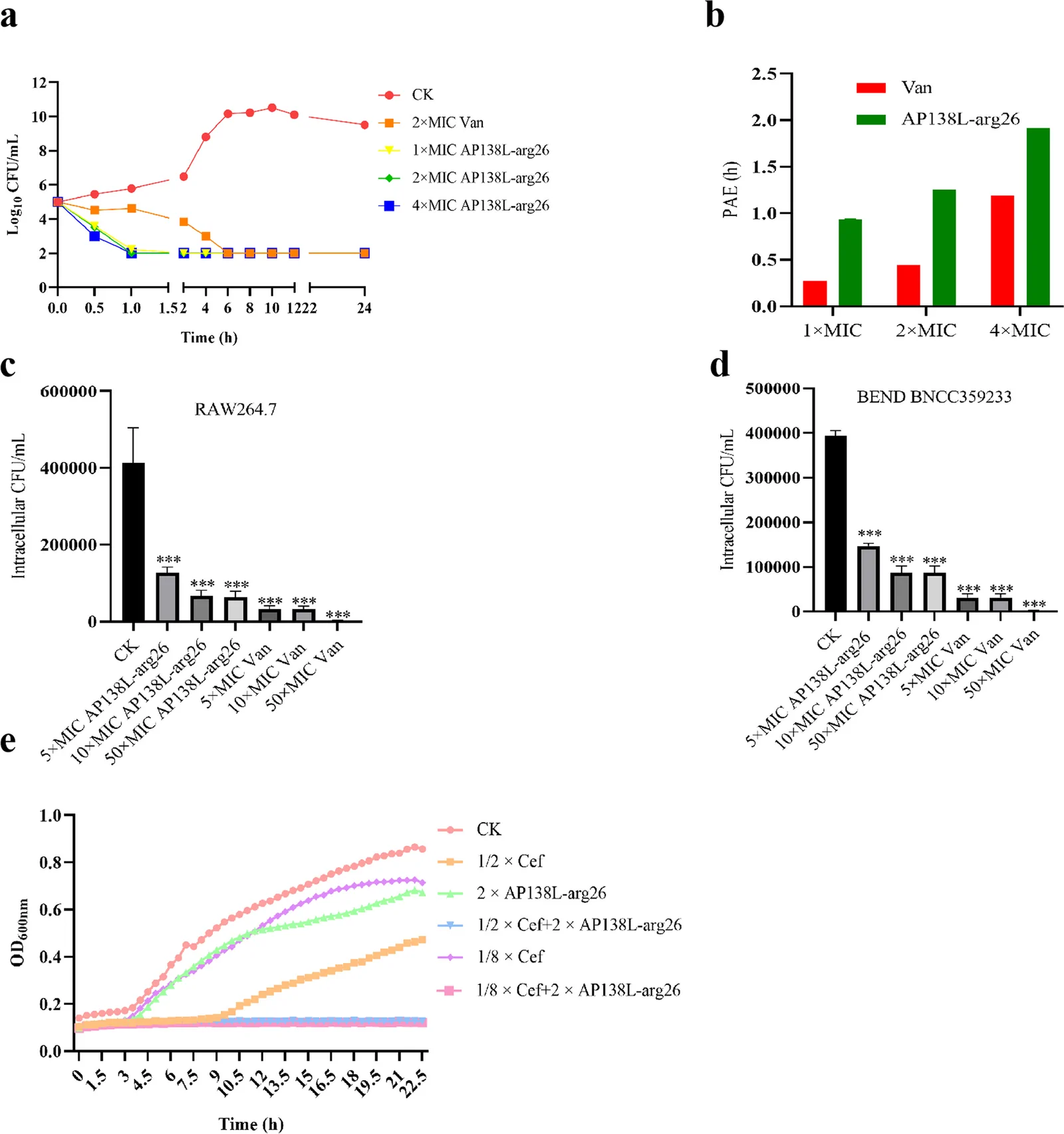
The pharmacodynamic evaluation of antimicrobial peptide AP138L-arg26 in extracellular and intracellular activity. **a** Time killing curve of AP138L-arg26 against *S. aureus* ATCC 43300, vancomycin (Van) as positive control, untreated group (CK) as blank control. **b** Post-antibiotic effect of AP138L-arg26 against standard *S. aureus* ATCC 43300, Van as control. Intracellular activity of AP138L-arg26 against *S. aureus* CVCC 546 in **c** mouse macrophage RAW 264.7 and **d** Bovine uterine epithelial cells (BEND). Asterisk (*) indicates the significance between control and treatment groups. ***P < 0.001. Results are expressed as the means from three biological replicates ± SD (*n* = 3). **e** Growth curve of *S. aureus* CVCC 546 after treatment with different combination of AP138L-arg26 and Ceftiofur Sodium (Cef)

As shown in Fig. 3a, all selected concentrations of AP138L-arg26 (1 × , 2 × , 4 × MIC) could kill 99.99% *S. aureus* ATCC 43300 within 1.5 h, while the antibiotic vancomycin needed at least 6 h. Meanwhile, AP138L-arg26 had longer PAE with 0.9 h, 1.25 h, and 1.91 h at 1 × , 2 × , 4 × MIC than vancomycin (0.27, 0.45, 1.18) (Fig. 3b). These results suggest that AP138L-arg26 had a faster and longer bactericidal effect than vancomycin, even within a low concentration (1 × MIC), which may be related to the different bactericidal mechanisms of AP138L-arg26 and vancomycin.

### Intracellular activity of AP138L-arg26 against *S. aureus*

As shown in Fig. 3c, d, AP138L-arg26 could kill intracellular *S. aureus* CVCC 546 in mouse macrophages RAW264.7 and bovine endometrial epithelial cells BNCC359233. The peptide of AP138L-arg26 could kill more than 70% of bacteria at a concentration of 5 × MIC, and the bactericidal rate was up to 85% at 50 × MIC. Therefore, AP138L-arg26 exhibited potent intracellular bactericidal ability, laying the foundation for in vivo applications.

### The synergism assay of AP138L-arg26 with antibiotic

As shown in Table 3, the FICI values of peptides AP138L-arg26 and vancomycin, ceftiofur sodium, ampicillin, or streptomycin sulfate ranged from 0.375 to 0.75, showing a synergistic effect. AP138L-arg26 had the lowest FICI value (0.375) with ceftiofur sodium. The bacterial growth curve results showed that AP138L-arg26 (8µg/mL, 2 × MIC) and sublethal levels of ceftiofur sodium alone (0.25 µg/mL, 1/8 MIC) had little effect on the growth of *S. aureus* CVCC 546 (Fig. 3e), but their combination sharply inhibited the growth of *S. aureus* CVCC 546. Therefore, the combination of AP138L-arg26 and ceftiofur sodium had the potential to be used to cure infection by *S. aureus*.

**Table 3:** Synergism of peptide AP138L-arg26 with antibiotics

Combination	Variety	*S. aureus* CVCC 546			
		MIC_a_ (µg/mL)	MIC_c_ (µg/mL)	FIC	FICI index
AP138L-arg26-vancomycin	AP138L-arg26	4	1	0.25	0.5
	Vancomycin	1	0.25	0.25	
AP138L-arg26-Streptomycin sulfate	AP138L-arg26	4	2	0.5	1
	Streptomycin sulfate	1	0.5	0.5	
AP138L-arg26-ceftiofur sodium	AP138L-arg26	4	0.25	0.0625	0.3125
	Ceftiofur sodium	1	0.25	0.25	
AP138L-arg26-ampicillin	AP138L-arg26	4	4	1	1.0625
	Ampicillin	0.125	0.0078	0.0625	

MICa, the MIC of peptide/antibiotic alone; MICc, the MIC of the peptide and antibiotic in combination

### Stability in vitro assay

These results show that AP138L-arg26 maintained its original MIC value (8 µg/mL) after incubation at different temperatures (20 °C, 40 °C, 60 °C, 80 °C), but the antibacterial activity was lost (> 64 µg/mL) under 100 °C. The antimicrobial activity of AP138L-arg26 was unaffected by gastric juice and different pH values (2–10). AP138L-arg26 was sensitive to trypsin, losting activity within 30 min after incubation (> 64 µg/mL) (Table 4), this would be the key limitation factor at least special for its oral administration.

**Table 4:** The stability of AMPs in different environments

AP138L-arg26 against *S. aureus* CVCC 546					
Condition	MIC	Condition	MIC	Condition	MIC
Heat (20 °C)	8	KCl (1.25 mM)	8	50% serum (0.5 h)	8
Heat (40 °C)	8	KCl (2.5 mM)	8	50% serum (1 h)	8
Heat (60 °C)	8	KCl (5 mM)	8	50% serum (2 h)	8
Heat (80 °C)	8	CaCl_2_ (1.25 mM)	8	50% serum (4 h)	8
Heat (100 °C)	> 64	CaCl_2_ (2.5 mM)	8	50% serum (6 h)	8
pH 2	8	CaCl_2_ (5 mM)	16	50% serum (8 h)	8
pH 4	16	MgCl_2_ (1.25 mM)	8	Gastric juice (0.25 h)	4
pH 6	16	MgCl_2_ (2.5 mM)	16	Gastric juice (0.5 h)	4
pH 8	8	MgCl_2_ (5 mM)	16	Gastric juice (1 h)	4
pH 10	16	25% serum (0.5 h)	8	Gastric juice (2 h)	8
NaCl (50 mM)	8	25% serum (1 h)	16	Intestinal juice (0.25 h)	> 64
NaCl (100 mM)	8	25% serum (2 h)	16	Intestinal juice (0.5 h)	
NaCl (150 mM)	8	25% serum (4 h)	8	Intestinal juice (1 h)	
NaCl (200 mM)	16	25% serum (6 h)	16	Intestinal juice (2 h)	
NaCl (300 mM)	16	25% serum (8 h)	8	Control (without treatment)	8

### Structure analysis

The secondary structures of AP138L-arg26 were measured in a different environment of 20 mM SDS and 50% TFE solution, which was used to simulate the hydrophobic environment of eukaryotic cells and the bacterial cell membrane environment, respectively. The results showed a positive peak at 196 nm and two negative peaks at 208 nm and 228 in 50% TFE solution, indicating an α/β spatial structure in these environments (Figure S2). However, the second structure of AP138L-arg26 had obviously changed (α-Helix, 4.72 to 6.06%; β-sheet, 47.78 to 42.82%; β-turn, 15.6 to 17.4%) in 20 mM SDS, especially for the increased α-Helix (1.34% increase), which could enhance membrane interactions between bacteria and AP138L-arg26 (Table S1).

### High safety of AP138L-arg26 in vitro and vivo

**Fig. 4:**
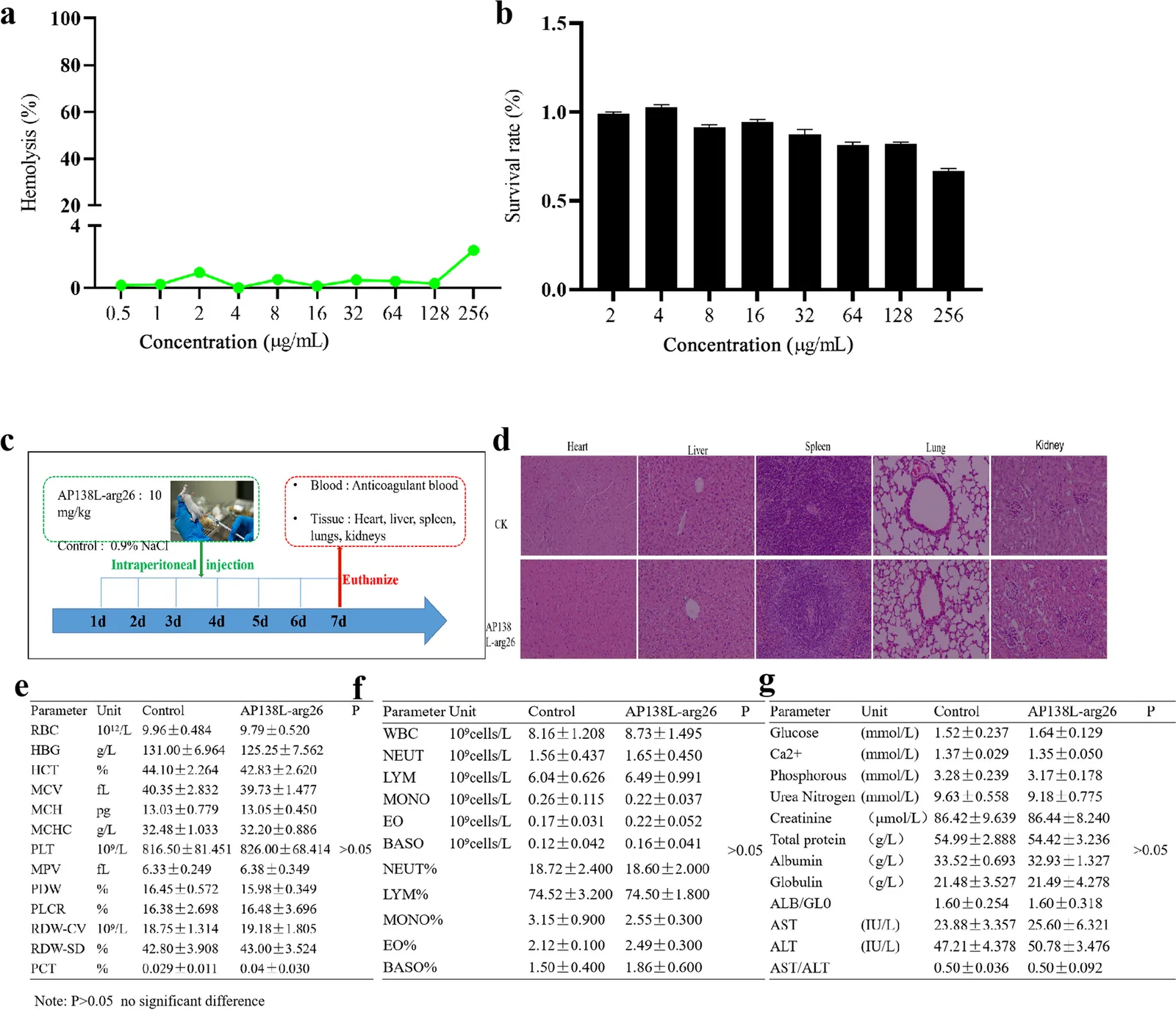
The safety of AP138L-arg26 in vitro and in vivo. **a** Hemolytic activity of AP138L-arg26 against fresh mouse red blood cells. **b** Cytotoxicity of the AP138L-arg26 against RAW264.7 cells. ICR female mice (*n* = 4) were intraperitoneally administered with AP138L-arg26 (10 mg/kg) daily for a week. **c**–**g** Histology images (H&E) stained (**d**), whole-blood cell profiles (**e**, **f**), serum biochemical 7 index (g) of mice after treatment with AP138L-arg26 for a week. CK, untreated group; scale bar, 100 µm

As shown in Fig. 4a, b, there was only 3% hemolysis and over 75% cell viability at a high concentration of AP138L-arg26 (256 µg/mL) in vitro, which indicated that AP138L-arg26 had low hemolysis and cytotoxicity. In vivo, mice (ICR, 6-8 weeks) were intraperitoneally injected with AP138L-arg26 at a concentration of 10 mg/kg (Fig. 4c–g). Compared with the control group, there were no significant differences in the whole blood detection indexes and biochemical indexes of the AP138L-arg26 treatment group. Moreover, the tissues of the AP138L-arg26 treatment group such as the heart, kidney, spleen, lung, and liver had no hyperemia, bleeding, hyperplasia, or other lesions in the full field, being almost same as those of the control group (Fig. 4d). These data indicate that the AP138L-arg26 peptide has excellent safety and good potential as clinical therapeutic drugs.

### The mechanism of AP138L-arg26 against *S. aureus* CVCC 546

#### SEM

**Fig. 5:**
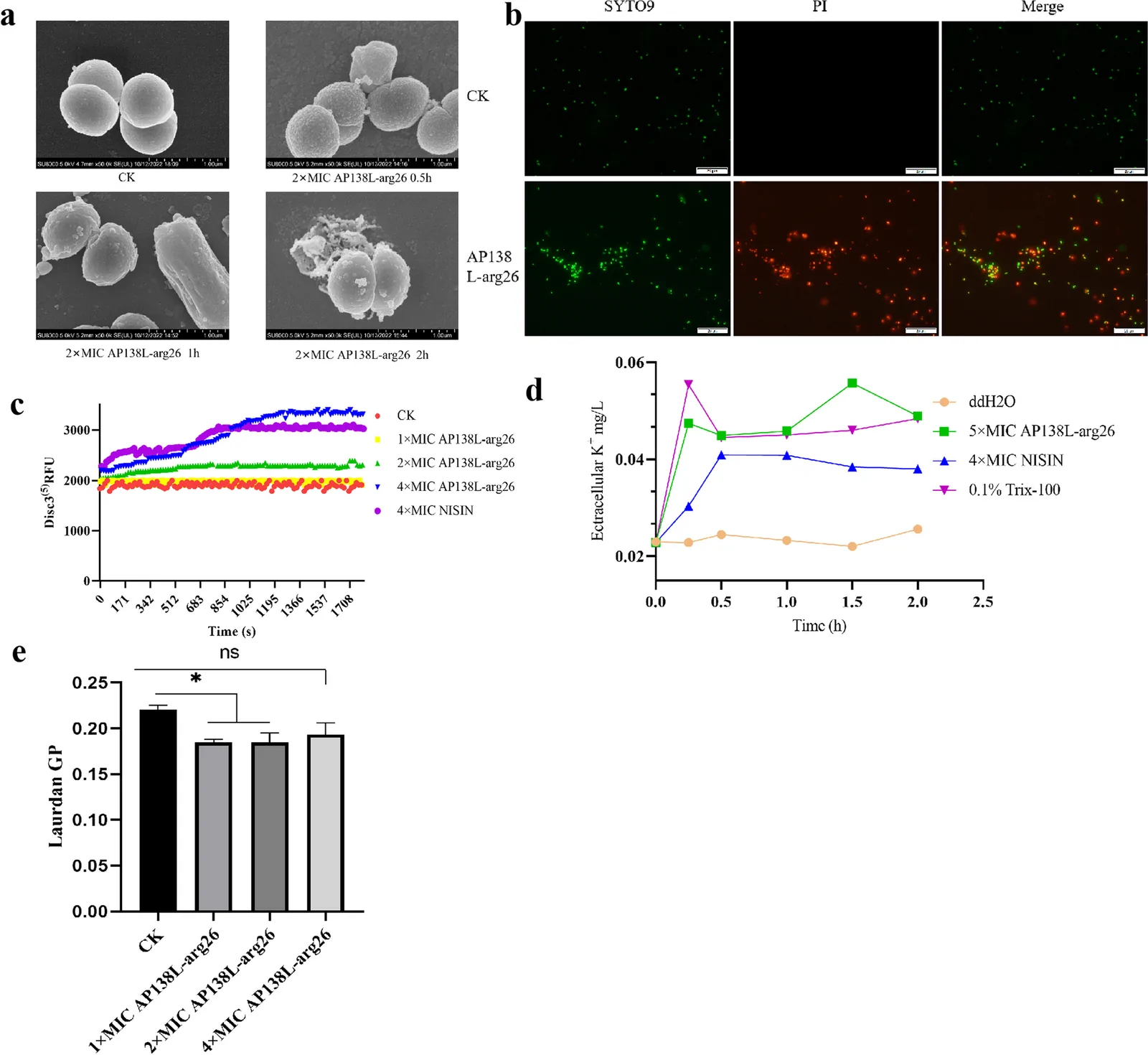
Study on the effect of AP138L-arg26 on cell membranes. **a** The morphological changes of *S. aureus* CVCC 546 were observed by SEM after AP138L-arg26 treatment. **b** The destruction of cell membranes by antimicrobial peptide AP138L-arg26 was observed by SYTO9/PI. **c** Detection of cell membrane potential after incubation of AP138L-arg26 with *S. aureus* CVCC 546. **d** The K^+^ leakage after incubation of AP138L-arg26 with *S. aureus* CVCC 546. **e** Membrane fluidity. Asterisk (*) indicates the significance between control and treatment groups, *P* < 0.05; (ns) indicates the no significance between control and treatment groups. CK, untreated group

The morphology changes of bacteria after treatment with AP138L-arg26 were observed using SEM. As shown in Fig. 5a, the surface of *S. aureus* CVCC 546 (nearly 100%) was smooth and spherical without peptide treatment. However, 60–80% of cell structures changed significantly with surface roughness, granular secretion, perforation, and deformation after treatment with 2 × MIC peptide AP138 at 0.5, 1, and 2 h. More than 80% of *S. aureus* CVCC 546 had significantly changed structure at 1 h. The results showed that AP138L-arg26 could destroy the structure of *S. aureus*.

### The effect of AP138L-arg26 on the cell membrane

According to the SEM results demonstrated that AP138L-arg26 caused disruption to the cell membrane or wall. Thus, a PI/SYTO9 assay was conducted to prove the destructive effect of AP138L-arg26 on *S. aureus* CVCC 546. The untreated group was almost stained green by SYTO9 (over 99%), demonstrating that they were predominantly living cells. The AP138L-arg26 treatment group could disrupt the cell membrane integrity with more than 50% cells stained red by PI (Fig. 5b). K^+^ leakage further illustrates the disruptive effect of AP138L-arg26 on bacterial membrane, the results showed that the AP138L-arg26 treated group significantly increased the extracellular K^+^ leakage level (0.05 mg/L) compared to the untreated bacteria (0.02 mg/L), similar to the positive control (0.1% TritonX-100) (Fig. 5d). It was also found that AP138L-arg26 could affect the membrane potential, as measured using DiSC_3_(5) fluorescence staining, the relative fluorescence units (RFU) values of the untreated and 1 × MIC AP138L-arg26-treated groups were around 2000, and the 4 × MIC AP138L-arg26 and nisin (positive control) groups were above 3000 (a 1000 increase) (Fig. 5c), which was mainly due to the depolarization caused by the destructive effect of the AP138L-arg26 on the bacterial membrane when the special compounds insert into lipid bilayers, they can change the membrane fluidity and disrupt the normal plasma membrane fluidity homeostasis, which further leads to leakage of cellular components and bacterial death. Therefore, a dye, Laurdan was used to measure the *S. aureus* membrane fluidity and quantified it using the Laurdan GP index. The results showed that the Laurdan GP of *S. aureus* decreased from 0.22 to 0.18 after treatment with 2 × MIC AP138L-arg26, which indicated an increase in the fluidity of the bacterial membrane due to the interaction of the AP138L-arg26 with the membrane (Fig. 5e). These results suggested that AP138L-arg26 could destroy the cell membrane of *S. aureus*.

### The effect of AP138L-arg26 on bacterial metabolism

**Fig. 6:**
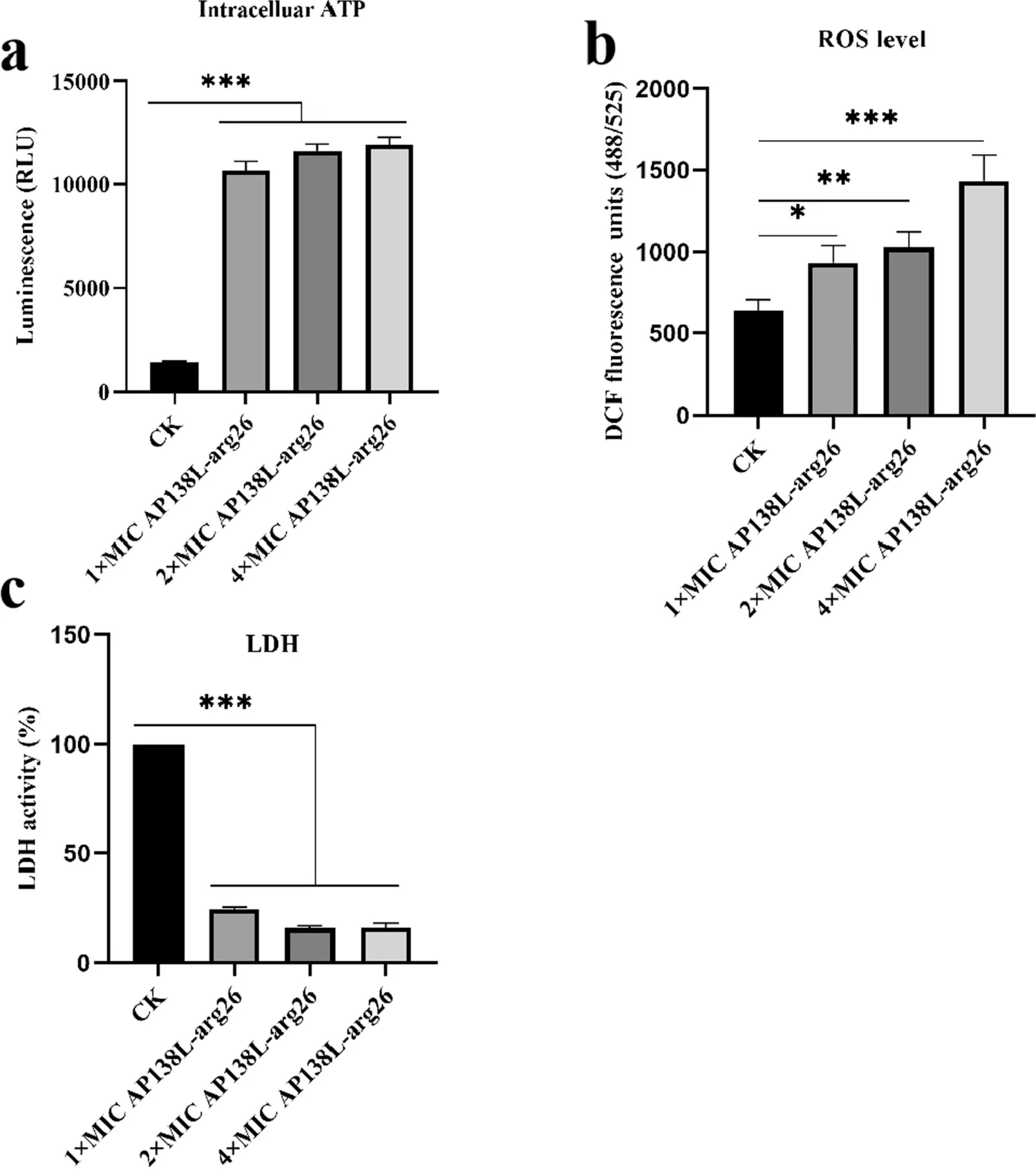
Effects of AP138L-arg26 on cell metabolism. **a** the level of Intracellular ATP, **b** the level of ROS, **c** the level of LDH. CK, untreated group. Asterisk (*) indicates the significance between control and treatment groups. ***P < 0.001. Results are expressed as the means from three biological replicates ± SD (*n* = 3)

In order to further study whether AP138L-arg26 could affect the metabolism to kill bacteria, bacterial respiration was detected using an ATP kit. The results showed that AP138L-arg26 could increase intracellular ATP: the RLU values of 1 × , 2 × , 4 × MIC AP138L-arg26-treated groups (10,700, 11,500, and 11,900, respectively) were 7.6, 8.2, and 8.5 times higher than that of the untreated group (1400), which indicated a concentration-dependent pattern (Fig. 6a). The increase in ROS causes a series of cascading reactions such as oxidation of lipids, proteins, and damage to DNA, inducing bacteria death. After treatment with 1 × , 2 × , and 4 × MIC AP138L-arg26, the fluorescence values increased from 600 (blank control, CK) to 930, 1000, and 1400 with concentration-dependent (Fig. 6b). LDH levels decreased after antimicrobial peptide incubation. The LDH activity % of CK, 1 × , 2 × , and 4 × MIC AP138L-arg26 were 100%, 24%, 16%, and 16% respectively, which indicated a concentration-dependent pattern (Fig. 6c). The results showed that AP138 could induce the ROS generation of *S. aureus* CVCC 546, ultimately promoting cell death and reducing the probability of resistance generation.

## Discussion

*S*. *aureus* is a pathogenic bacterium that greatly threatens human health and the production efficiency of livestock and poultry. Many bacteria have developed drug resistance, particularly MRSA (Pinto et al. 2019). The rise of drug resistance has triggered a public health crisis; therefore, there is an urgent need to develop new antibacterial agents to alleviate bacterial resistance (Peters et al. 2010; Li et al. 2020). The unique bactericidal mechanism and multiple functions of AMPs have been extensively studied as antibiotic substitutes (Rao 1995; Peters et al. 2010). Among them, plectasin is the first fungal defensin extracted from *Pseudoplectania nigrella (Saprophytic ascomycetes)*; it kills G^+^ bacteria but has low activity and some cytotoxicity. Therefore, researchers have conducted in-depth studies on the peptides derived from plectasin (Mygind et al. 2005; Schneider et al. 2010). AP138 is a derived peptide with potential for clinical development, obtained by chemical synthesis due to the D type Arg at the 26th position of the sequence (Stecher et al. 2014; Groo et al. 2018). In this work, natural amino acid sequence (L-type amino acid) was obtained using heterologous expression to reduce the high cost of chemical synthesis, and the activity and bactericidal mechanism of the L-type AP138 were analyzed.

As can be seen in Table 1, it was predicted that AP138L-arg26 would have a high charge (+4.5) and antimicrobial peptide potential. Compared with plectasin, the substitution of five amino acids (D9S, M13L, Q14R, N17R, and K26R) significantly changed the physicochemical properties of AP138L-arg26 including an increased positive charge (+1 to  +4.5) and hydrophobicity (32 to 33%) (Table 1), making it a good AMP possibility. Although D-type modification of the Arg in the sequence (D-type AP138) could effectively improve the tolerance of the antimicrobial peptide to trypsin which this property cannot be maintained in our AP138L-arg26, its chemical synthesis is difficult on a large scale and at low cost, increasing the challenges and difficulties of clinical development (Stecher et al. 2014; Umerska et al. 2016; Groo et al. 2018; Koo and Seo 2019. Heterologous expression can achieve high yields, thus reducing the cost of developing the target protein. The exogenous expression technology of proteins in a y*east* expression system or *E. coli* expression system is relatively mature, but the expression of small molecule peptides (< 50 aa) is relatively difficult execpt few successful cases from fungal defensins and others (Zhang et al. 2011; Zhang et al. 2014; Cao et al. 2015; Yang et al. 2019; Shen et al. 2021; Gries 2022; Zeng et al. 2022). In addition, the AMPs Retrocyclin-101, and Protegrin-1, and Abaecin was also expressed in a prokaryotic expression system with a low yield (Lee et al. 2011). More details in our previous works, many antimicrobial peptides were successfully expressed in *P. pastoris* with high yield at level of  1.0-3.0 g/L (supernatant) and even higher level via high-density fermentation such as NZ2114, NZX, ID3 and NZL, with continous optimisation including enhancing the microbial biomass, controlling the fermentation and induction conditions, the increasing production capacity of equipment per unit volume and modificating expression system (Zhang et al. 2014. Liu et al. 2020; Feng et al. 2012; Li et al 2020; Shen et al. 2021; Hao et al. 2023; Jin et al. 2023). In this study, the recombinant vector AP138L-arg26 was successfully constructed and expressed for the first time at high levels in *P. pastoris*. The total protein concentrations of the fermentation supernatant and microbial biomass were up to 3.1 mg/mL (95% purity) and 0.36 g/mL, respectively, after 120 h high density fermentation in a 5-L fermenter (Fig. 2), higher than those of NZ2114 (2.390 mg/mL, 94.8% purity) (Zhang et al. 2014), and the production cost of AP138L-arg26 was significantly lower than that of chemical synthesis (solid phase).

The MIC is one of the key indicators for the early screening of active AMPs, for which values of MICs under 16 µg/mL may be clinically relevant (Rao 1995; Patel et al. 2009; Oh et al. 2019). The MICs of AP138L-arg26 were 2–16 µg/mL (0.45–3.6 µM) against selected standard and clinical *S. aureus*, *S. epidermidis*, *S. dysgalactiae*, and *S. agalactiae* (Table 2). Although the MIC values were lower than those of D-type AP138 (0.125–4 µg/mL), it is still superior to lincomycin, which is mainly used clinically against G^+^ bacteria. The structure and function of AMPs are closely related, and in-depth investigation of the structure-activity relationship is an essential step in studying AMPs (Rost and Sander 1994; Rao 1995). It was analyzed that AP138L-arg26 has a special CSαβ structure with  +4.5 net charge and 33% hydrophobic ratio in a normal environment with ddH_2_O. In the simulated bacterial membrane structure (20 mM SDS), AP138L-arg26 showed significant secondary structure changes, increased α-helix structure (4.72 to 6.06%), which may be related to the bactericidal function of the peptide, while it does not change significantly in the simulated eukaryotic cell membrane structure (50% TFE buffer), suggesting no damage to cells (Figure S2). The killing curves showed that 1 × , 2 × , 4 × MIC of AP138L-arg26 could kill all *S. aureus* ATCC 43300 within 1.5 h, which was shorter than that of vancomycin (6 h) (Fig. 3a). These results showed that the AMP AP138L-arg26 could rapidly killed bacteria. The PAE is an important guide for the rational use of clinical drugs, and the re-evaluation of adverse effects of antibiotics and combination drugs. In this study, the PAE of AP138L-arg26 was longer than that of antibiotics (Fig. 3b), indicating that low doses and long dosing intervals are feasible, which could reduce the amount of drugs used and alleviate the problem of drug resistance caused by the misuse of antibiotics (Li et al. 2020). *Staphylococcus* pathgens can evade antibiotics by entering cells (Wang et al. 2018). AP138L-arg26 had the ability to enter mammalian cells and can kill infected bacteria within cells such as *S. aureus* CVCC 546 in mouse macrophages RAW264.7 and bovine uterine epithelial cells in this study (Fig. 4c, d). Stability and safety are the factors that must be controlled when drugs enter the clinic, as high toxicity and low stability will hinder AMPs’ application (Koo and Seo 2019). AP138L-arg26 retained its antimicrobial activity in different concentrations of salt ions, pH, temperature, and pepsin, but it was sensitive to trypsin and lost antimicrobial activity for 30 min. These results showed that it remained the antimicrobial activity (MIC against *S. aureus* ATCC 43300, 8 µg/mL) and has the potential for topical drug development (Table 2), whereas it may need to be encapsulated for oral administration (Umerska et al. 2017; Groo et al. 2018). AP138L-arg26 had a better safety rating in vitro and in vivo, as reflected in the high cell survival (over 75% for mouse macrophages RAW264.7) and the low hemolysis (less than 3%) at a high concentration of 256 µg/mL of AP138L-arg26 in vitro (Fig. 4a, b). After intraperitoneal injection of 10 mg/kg AP138L-arg26 for 1 week, it was found that there were no obvious differences in whole blood detection indexes (< 4.5%), biochemical index (< 4%). The histological sections of the heart, liver, spleen, lung, and kidney were not damaged and retained a normal structure (no bleeding, hyperemia, or hyperplasia) indicating the safety of AP138L-arg26 in vivo (Fig. 4c–g). These results indicate that AP138L-arg26 has a high safety profile as a drug in *vivo* and in *vitro*.

In general, the researchers suggest that the unique positive charge and hydrophobic properties of most AMPs could interact with bacterial cell membranes and exert bactericidal functions (Gao et al. 2021; Pinto et al. 2019; Zheng et al. 2022). Changes in bacterial morphology were first observed using SEM after treatment with 2 × MIC AP138L-arg26 for 1 h, and most bacteria had rough surfaces, depressions, and granular secretions (Fig. 5a). It was further shown that AP138L-arg26 had a directly destructive effect on the cell membrane through PI staining, K^+^ leakage, and membrane fluidity assays (Fig. 5a–e). AP138L-arg26 might mainly be considered to interact with the negatively charged components on the surface of the bacterial membrane through itself charges, and then insert into the cell membrane, interfering with its orderly arrangement, causing further destruction (She et al. 2022; Wang et al. 2021a, b). This was similar to the case with the antimicrobial peptide 5j and L007-0069 (She et al. 2022). AP138L-arg26 also affect the bacterial metabolism: (i) After the incubation of AP138L-arg26 with bacteria, intracellular ATP levels were elevated (maximum: 8.5-fold), which may cause bacteria to switch from a dormant state to an active state that is more conducive to them being killed (Shi et al. 2022). (ii) Intracellular ROS were increased twofold with 2 × MIC of AP138L-arg26, which suggests that bacteria could be damaged through bacterial auto-oxidation (Shi et al. 2022), this is an important way in which drugs could damage bacteria. (iii) At the same time, the amount of LDH decreased (lowest, 16%), which may limit the level of respiratory metabolism due to LDH being an essential enzyme in the respiratory chain (Wang et al. 2021a, b) (Fig. 6).  All in all, AP138L-arg26 may kill bacteria by damaging cell membranes and influencing bacterial metabolism (ATP, ROS, LDH).

Finally, we changed the D-type AP138D to L-type AP138L-arg26, and it was successfully expressed in *P. pastoris* with high production. It was demonstrated that AP138L-arg26 had a faster and longer bactericidal effect compared with conventional antibiotics, high stability, and excellent safety in vivo and in vitro. It was revealed that AP138L-arg26 has multiple bactericidal mechanisms including membrane rupture and metabolism imbalance, which is also an important reason that antimicrobial peptides do not tend to develop resistance. The better derived AMPs of plectasin will be worth of developing in the future so that the excellent property of high resistance to trypsin hydrolysis in AP138 construct could be merged and maintained in new AMP derivative with a high expression level.

## Supplementary Information

Below is the link to the electronic supplementary material.

Supplementary file1 (PDF 272 KB)

## Data Availability

All data generated or analyzed during this study are included in this published article (and its supplementary information files).
