# Supplementary material for: Expression and characterization of the new antimicrobial peptide AP138L-arg26 anti Staphylococcus aureus
Source: Appl Microbiol Biotechnol. 2024 Jan 13;108(1):111. doi: 10.1007/s00253-023-12947-w (PMC10787893; doi:10.1007/s00253-023-12947-w)
Supplement: Supplementary file 1 — Supplementary file1 (PDF 272 KB) [file 253_2023_12947_MOESM1_ESM.pdf]

Applied Microbiology and Biotechnology

Supplementary Material

Expression and characterization of the new Antimicrobial peptide AP138L-arg26 anti *Staphylococcus aureus*

Kun Zhang[1,2,3], Na Yang[1,2,3*], Da Teng[1,2,3], Ruoyu Mao[1,2,3], Ya Hao[1,2,3], Jianhua Wang[1,2,3*]

[1] Gene Engineering Laboratory, Feed Research Institute, Chinese Academy of Agricultural Sciences, Beijing 100081, P. R. China

[2] Innovative Team of Antimicrobial Peptides and Alternatives to Antibiotics, Feed Research Institute, Chinese Academy of Agricultural Sciences, Beijing 100081, P. R. China;

[3] Key Laboratory of Feed Biotechnology, Ministry of Agriculture and Rural Affairs, Beijing 100081, P. R. China

* Corresponding author

Ph.D. Na Yang, nana_891230@126.com

Prof., Ph.D., and senior PI. Jianhua Wang, and postal address of all authors as:

Gene Engineering Laboratory, Feed Research Institute, Chinese Academy of Agricultural Sciences

No. 12 ZhongguancunNandajie St., Haidian District, Beijing 100081, P. R. China

E-mail address: wangjianhua@caas.cn; wangjianhua.peking@qq.com

Phone: 0086-10-82106081, 0086-10-82106079; Fax: 0086-10-82106079

Table S1 the second structure of AP138L-arg26

peptides	α-Helix	β-sheet	β-turn	coli
plectasin	30.75	20.8	95.8	12.5
AP138L-arg26	20.8	12.5	120.8	12.5

Note: All data were predicted by online helix-turn-helix software.

Figure Legends

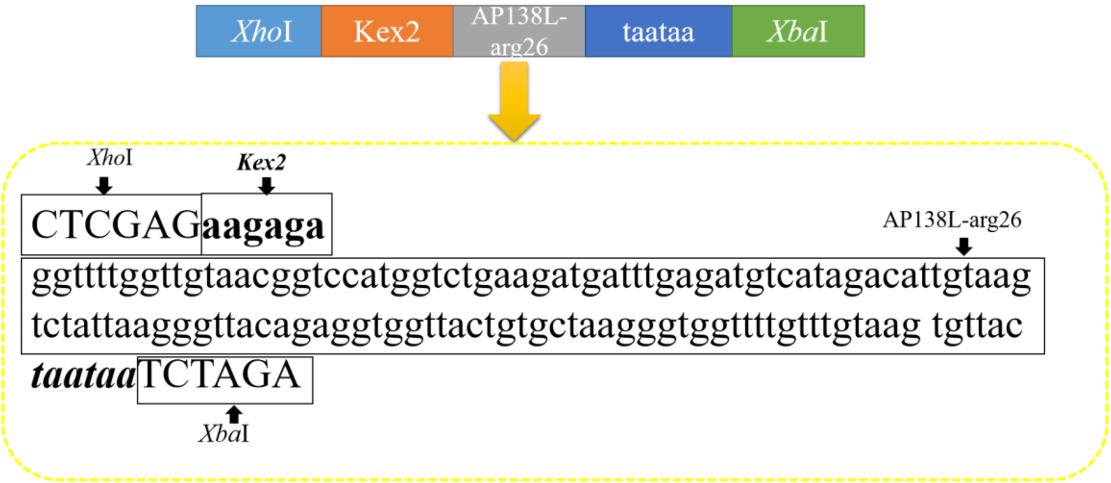

Figure S1 The schematic and codon-optimized nucleotide sequences of the AP138L-arg26 genes

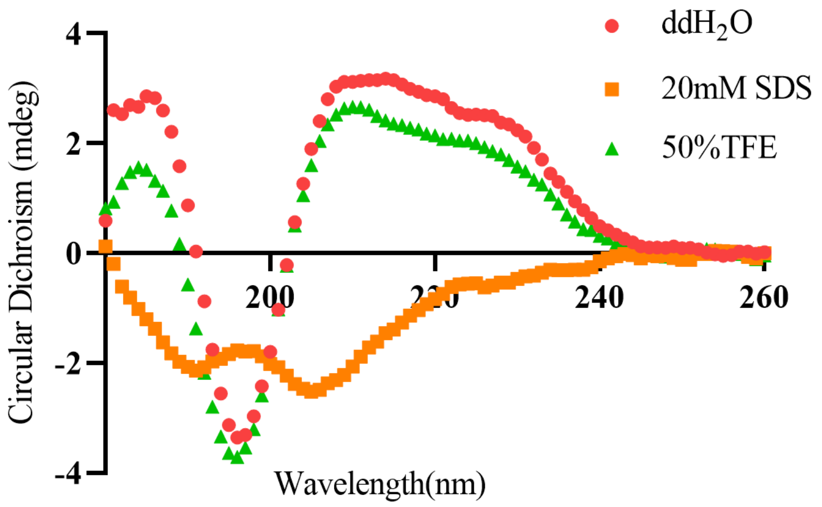

Figure S2. CD spectra of the peptide AP138L-arg26 in H_2O, 20 mM SDS, or 50% TFE.
